# Targeting of Tomato Bushy Stunt Virus with a Genetically Fused C-End Rule Peptide

**DOI:** 10.3390/nano13081428

**Published:** 2023-04-21

**Authors:** Luca Marchetti, Lorena Simon-Gracia, Chiara Lico, Mariateresa Mancuso, Selene Baschieri, Luca Santi, Tambet Teesalu

**Affiliations:** 1 Laboratory of Biomedical Technologies, Italian National Agency for New Technologies, Energy and Sustainable Economic Development, ENEA, Casaccia Research Center, Via Anguillarese 301, 00123 Rome, Italy; 2 Department of Agriculture and Forest Sciences (DAFNE), University of Tuscia, Via S. Camillo De Lellis, 01100 Viterbo, Italy; 3 Laboratory of Precision and Nanomedicine, Institute of Biomedicine and Translational Medicine, University of Tartu, Ravila 14b, 50090 Tartu, Estonia; 4 Laboratory of Biotechnologies, Italian National Agency for New Technologies, Energy and Sustainable Economic Development, ENEA, Casaccia Research Center, Via Anguillarese 301, 00123 Rome, Italy; 5 Materials Research Laboratory, University of California, Santa Barbara, CA 93106, USA

**Keywords:** Tomato Bushy Stunt Virus, plant virus nanoparticles, neuropilin-1, C-end rule, homing peptide, drug delivery systems

## Abstract

Homing peptides are widely used to improve the delivery of drugs, imaging agents, and nanoparticles (NPs) to their target sites. Plant virus-based particles represent an emerging class of structurally diverse nanocarriers that are biocompatible, biodegradable, safe, and cost-effective. Similar to synthetic NPs, these particles can be loaded with imaging agents and/or drugs and functionalized with affinity ligands for targeted delivery. Here we report the development of a peptide-guided Tomato Bushy Stunt Virus (TBSV)-based nanocarrier platform for affinity targeting with the C-terminal C-end rule (CendR) peptide, RPARPAR (RPAR). Flow cytometry and confocal microscopy demonstrated that the TBSV-RPAR NPs bind specifically to and internalize in cells positive for the peptide receptor neuropilin-1 (NRP-1). TBSV-RPAR particles loaded with a widely used anticancer anthracycline, doxorubicin, showed selective cytotoxicity on NRP-1-expressing cells. Following systemic administration in mice, RPAR functionalization conferred TBSV particles the ability to accumulate in the lung tissue. Collectively, these studies show the feasibility of the CendR-targeted TBSV platform for the precision delivery of payloads.

## 1. Introduction

Nanocarriers are increasingly applied for the delivery of imaging reagents and drugs to specific cells and tissues. Besides widely used synthetic nanocarriers, self-assembled protein nanoparticles (NPs) [1] and virus-like particles (VLPs) [2] are developed as drug delivery systems. Each platform presents a unique set of advantages and limitations in terms of immunogenicity, toxicity, pharmacokinetics, and targeting specificity. Plant viruses, generated by self-assembly of the viral coat protein (CP) subunits [3], are safe, biocompatible, biodegradable NPs and can be cost-effectively produced in plants [4]. The surface of the plant viruses can be functionalized with chemically conjugated or genetically fused peptides for affinity targeting (in the case of particles loaded with diagnostic or therapeutic cargoes), or for the display of foreign immunogenic epitopes in vaccine applications [5,6,7]. Cowpea Mosaic Virus (CPMV) [8,9], Tobacco Mosaic Virus (TMV) [10,11], and Potato Virus X (PVX) [12,13] are examples of plant viruses widely used as nanoparticles (plant virus nanoparticles (pVNPs)) for nanotechnology applications. Due to their elongated shape, TMV and PVX particles are poorly suited as payload carriers; instead, TMV- and PVX-based systems are applied for the molecular fabrication of nanodevices [11], or for the display of immunogenic epitopes [12]. Whereas CPMV-derived spherical pVLPs can be effectively loaded with cargo [14], CPMV targeting with surface-homing peptides remains challenging due to instability and proteolytic processing of the exogenous peptides fused to the viral coat protein [15]. Tomato Bushy Stunt Virus (TBSV), the prototypic member of the Tombusviridae family, provides a pVNP scaffold for the stable C-terminal display of peptides [16], and spherical TBSV NPs can be efficiently loaded with cargo molecules [17]. The structure of TBSV has been resolved at atomic resolution [18], with the 30 nm capsid composed of 180 identical copies of a single coat protein (CP/p41) arranged in T = 3 symmetry [19]. The CP includes the RNA binding domain (R), the shell domain (S) forming the capsid backbone, and the C-terminal protruding (P) domain that can accommodate exogenous peptides for the display [20]. In a proof-of-concept study, systemically administered TBSV NPs guided by genetically fused tumor-homing peptides showed tropism towards medulloblastoma lesions. In the case of TBSV NPs loaded with doxorubicin, this targeting translated into increased anticancer activity [17]. Active targeting of drugs and NPs to improve their efficacy and lower off-target toxicity relies on specific binding of affinity ligands such as peptides, aptamers, and antibodies to specific systemically accessible molecular markers expressed in a target tissue [21,22]. Peptides that target normal and diseased tissues are typically identified by in vivo screening of phage libraries in live mice [23,24]. The power of in vivo phage screening is illustrated by the discovery of tumor-penetrating peptides (TPPs), including the clinical-grade iRGD peptide [25]. These peptides activate an endocytic transport pathway related to but distinct from macropinocytosis through a three-step process that involves binding to a primary tumor-specific receptor, a proteolytic cleavage, and binding to a second receptor, NRP-1, to activate the transport pathway [26]. The critical element in all TPPs is the presence of the cryptic R/KXXR/K motif able to interact with cell and tissue penetration receptor NRP-1 only when exposed at the C-terminus [27]. CendR receptor NRP-1 is widely expressed in normal tissues and overexpressed in malignant and malignancy-associated cells in a wide range of solid tumors, including prostate, breast, pancreas and hepatocellular carcinoma, melanoma, glioblastoma, and leukemia [28,29,30,31]. NRP-1 targeting with prototypic CendR peptide RPARPAR (shortened RPAR) is a simple and robust system that can be used for optimization of homing peptide-guided NP delivery [32,33,34,35]. Here we report the development of engineered TBSV displaying RPAR peptides and establish the ability of the fluorophore-labeled TBSV pVNPs to specifically target NRP-1-positive cells in vitro and in vivo. We also demonstrate that TBSV-RPAR loaded with the widely used anthracycline doxorubicin (DOX) exert NRP-1-dependent toxicity on cultured cancer cells. These proof-of-concept studies show the feasibility of a CendR-targeted TBSV-based delivery platform.

## 2. Materials and Methods

### 2.1. Genetic Engineering of TBSV and Its Production in Plants

TBSV is a positive-sense single-stranded RNA virus with a linear genome of ~4800 nucleotides. To generate TBSV-RPAR, first, the DNA sequence encoding the RPARPAR peptide was inserted at the 3′-end of the *p41* gene of the plasmid vector covering the full viral genome, pTBSV-WT, as reported in [16]. Briefly, codon-optimized in vitro annealed DNA oligos encoding the peptide with *ApaI*/*PacI*-compatible ends (5′ CGGAGGTAGACCAGCTAGGCCTGCAAGATGAGAGCTCTTAAT 3′, 5′ TAAGAGCTCTCATCTTGCAGGCCTAGCTGGTCTACCTCCGGGCC 3′) were ligated into the properly double-digested plasmid generating the pTBSV-RPAR. *XmaI*-linearized pTBSV-WT and pTBSV-RPAR DNAs were transcribed in vitro using the MEGAscript T7 High Yield Transcription kit (Ambion Applied Biosystems, Waltham, MA, USA) to generate infectious RNA molecules corresponding to the viral genome. Virus RNA was used to inoculate 6–8-week-old *Nicotiana benthamiana* plants, grown under controlled conditions (24 °C, 16 h light/8 h dark) in a containment greenhouse, by abrading the adaxial side of 2 leaves/plant with carborundum (silicon carbide, VWR International, Radnor, PA, USA). TBSV NPs were purified from infected plants according to a previously developed protocol [16].

### 2.2. Chemical Conjugation of CF555 Dye on TBSV NPs

TBSV-WT^CF555^ and TBSV-RPAR^CF555^ NPs were generated by coupling CF555-maleimide (Sigma-Aldrich, Saint Louis, MO, USA) to exposed cysteine residues on the virus capsid. TBSV-WT and TBSV-RPAR at a concentration of 1 mg/mL in KP buffer (0.1 M potassium phosphate buffer pH 7.0) were mixed with 2000 molar excess of CF555-maleimide in DMSO per 1 TBSV particle (~10 CF555 molecules/CP unit) [14]. The final DMSO concentration was adjusted to 10% (*v*/*v*). The reaction was left to proceed at RT with agitation in the dark overnight. After labeling, the TBSV NPs were purified by ultracentrifugation over a 25% (*w*/*v*) sucrose cushion at 28,000 rpm using an SW 60 Ti rotor (Beckman Coulter Inc., Brea, CA, USA) for 3 h. The TBSV pellet was resuspended in 0.1 M potassium phosphate buffer pH 7. Virus concentration and number of conjugated dyes were determined by UV/Visible spectroscopy using a Nanodrop 2000c spectrophotometer (Thermo Scientific, Waltham, MA, USA) using the Lambert–Beer law considering the TBSV and CF555 molar extinction coefficients (4.5 mL mg^−1^ cm^−1^ at 260 nm and 150,000 M-1 cm^−1^ at 555 nm, respectively). For SDS-PAGE, denatured TBSV samples (4 µg per lane) were loaded on 4–20% Tris-glycine gels (Bio-Rad) and ran in Tris-glycine buffer (25 mM Tris, 192 mM glycine, 0.1% SDS, pH 8.3). Gels were imaged using a Li-Cor Odyssey M scanner. A fluorescence channel (520 nm) was used to detect CF555 TBSV NPs, and a colorimetric channel (630 trans) was used after staining with Coomassie Blue.

### 2.3. Enzyme-Linked Immunosorbent Assay (ELISA)

TBSV-RPAR/NRP-1 binding was assessed by ELISA. The high-binding polystyrene multiwell plates were coated with 2 µg/mL of recombinant NRP-1 (NRP-1-WT) receptor or with b1/b2 mutated NRP-1 (NRP-1-Mut) in 100 µL of phosphate-buffered saline (PBS, pH 7.4) at 4 °C overnight. Wells were washed and blocked with 5% low-fat milk in PBS at 37 °C for 2 h. After blocking, the wells coated with NRP-1-WT were incubated with 8 µg/mL of in-house developed mouse monoclonal antibody [36] at 37 °C for 30 min, and then TBSV-WT or TBSV-RPAR samples in PBS (100 µL at 1.5 µg/mL) were added to each well and incubated at 37 °C for 2 h. Wells were washed with 0.1% (*v*/*v*) Tween-20 in PBS to remove free TBSV NPs. The polyclonal rabbit anti-TBSV antibody (Agdia, Elkhart, IN, USA) diluted at 1:200 in PBS containing 2% low-fat milk was incubated at 37 °C for 90 min. The secondary antibody (polyclonal HRP-Donkey anti-rabbit IgG, Biolegend, San Diego, CA, USA) was used at a dilution of 1:3000 in PBS containing 2% low-fat milk and incubated at 37 °C for 1 h. TMB Reagent (3,3′,5,5′-tetramethylbenzidine, 1-Step Ultra TMB-Elisa Thermo Scientific, Waltham, MA, USA) was added to the wells, the colorimetric reaction was stopped with 100 μL of stop solution (2 M H_2_SO_4_), and the signal was measured at 450 nm using a microplate reader (Tecan, Männedorf, Swiss).

### 2.4. Flow Cytometry

PPC-1 human prostate carcinoma cells and M21 human melanoma cells were grown in DMEM containing 4.5 g/L glucose without L-Gln (Lonza, Basel, Swiss) supplemented with 10% fetal bovine serum (Gibco), 100 U/mL penicillin, and 100 µg/mL streptomycin (Gibco, Waltham, MA, USA). Cells were grown in the presence of 5% CO_2_ in a humidified environment at 37 °C. M21 and PPC-1 cells were seeded in a 24-well plate at 100,000 cells/well and grown overnight. TBSV-WT^CF555^ or TBSV-RPAR^CF555^ NPs (10 μg/well) were added to the cells and incubated at 37 °C for 1 h, washed three times with PBS, detached with non-enzymatic cell dissociation solution (Cellstripper, Corning, New York, NY, USA) and collected in a 1.5 mL tube. Cells were subsequently washed, resuspended in PBS, and analyzed using a BD Accuri C6 plus flow cytometer. Triplicates of each sample were examined, and 10,000 events (gated for live cells) were recorded. Data were analyzed using the BD Accuri C6 software. For the NRP-1 blocking, incubation with TBSV NPs (5 μg/well) was preceded by incubation with an in-house generated mouse anti-human mAb (at 10 µg/mL and RT, 30 min).

### 2.5. Confocal Imaging of Cultured Cells

For confocal microscope imaging, 100,000 cells/well in 500 μL of DMEM were seeded on glass coverslips in 24-well plates. TBSV-WT^CF555^ and TBSV-RPAR^CF555^ NPs (10 μg/well) were added, and the mixtures were incubated at 37 °C for 1 h. Cells were washed 3 times with PBS to remove unbound NPs and then fixed with 4% paraformaldehyde in PBS at RT for 10 min. Cell nuclei were stained with DAPI (1 ug/mL) at RT for 8 min, and then the coverslips were mounted in aqueous mounting medium Fluoromount-G (Electron Microscopy Sciences, Hatfield, PA, USA). For the PPC-1-GFP/M21 co-culture experiment, the mixed suspension of cells (100,000/well) was seeded on a glass coverslip as mentioned above and exposed to TBSV-RPAR^CF555^ NPs (10 µg/well) at 37 °C for 1 h. After fixation with 4% paraformaldehyde in PBS at RT for 10 min, the cells were incubated with blocking buffer (5% *w*/*v* BSA, 5% *v*/*v* FBS, and 5% *v*/*v* goat serum in PBS with 0.05% Tween-20) at RT for 30 min, followed by incubation with a primary in-house generated rabbit anti-human p32 antibody (diluted 1:200 in 0.2× blocking buffer) at RT for 30 min; then, they were washed and incubated with goat anti-rabbit Alexa 647-conjugated antibody (Invitrogen, Waltham, MA, USA) diluted 1:200 in 0.2× blocking buffer at RT for 30 min. After cell nuclei staining, the coverslips were mounted on glass slides using Fluoromount-G. For the pulse-chase co-localization experiment, cells were incubated with TBSV-RPAR NPs (10 μg/well) at 37 °C for 30 min, washed 3 times with fresh medium to remove unbound NPs, and chased for 30, 90, and 270 min. After chasing, cells were fixed in 4% paraformaldehyde in PBS pH 7.2 at RT for 10 min; then, they were permeabilized with 0.2% Triton-X 100 in PBS at RT for 10 min, washed 3 times in PBS, and incubated with blocking buffer at RT for 30 min. TBSV NPs were stained using rabbit anti-TBSV antibody (Agdia, Elkhart, IN, USA) diluted 1:200 in 0.2× blocking buffer at RT for 30 min followed by a goat anti-rabbit Alexa Fluor 546-conjugated secondary antibody (Invitrogen, Waltham, MA, USA) diluted 1:200 in 0.2× blocking buffer at RT for 30 min. Endolysosomes were identified using mouse anti-human CD107a (LAMP-1) antibody (Biolengend, San Diego, CA, USA) diluted 1:200 in 0.2× blocking buffer at RT for 30 min followed by Alexa Fluor 647-conjugated goat anti-mouse antibody (Invitrogen) diluted 1:200 in 0.2× blocking buffer at RT for 30 min. Finally, cell nuclei were stained using DAPI (1µg/mL) at RT for 8 min, and coverslips were mounted on glass slides using Fluoromount-G. An Olympus FV1200MPE confocal microscope (Olympus Europa SE & Co. KG, Hamburg, Germany) was used for imaging. Acquired images were analyzed using FluoView FV10-ASW 4.0 software (Olympus Europa SE & Co. KG). For co-localization analysis, Pearson’s R correlation coefficient using Fiji ImageJ Coloc 2 plugin (https://imagej.net/plugins/coloc-2, accessed on March 2022) was used. For each time point, ten different images were analyzed.

### 2.6. Doxorubicin Loading of TBSV NPs

Approximately 1 mg/mL of purified TBSV NPs were incubated in swelling buffer (0.1 M Tris, 50 mM EDTA, pH 8.0) at RT in agitation for 1 h. A 5000 molar excess of DOX (0.324 µg DOX/µg virus) was added, followed by incubation on a bench-top tube rotator at 4 °C overnight. To re-seal the virus cages, the samples were mixed in association buffer (0.2 M Na Acetate, 25 mM CaCl_2_, 25 mM MgCl_2_, pH 5.2) at RT for 1 h. DOX excess was removed by ultracentrifugation on a 25% sucrose cushion using an SW 60 Ti swing-out rotor (Beckman Coulter Inc., Brea, USA) at 28,000 rpm at 4 °C for 3 h. Pellets were resuspended in association buffer overnight and stored at 4 °C until needed. TBSV^DOX^ NPs were analyzed by UV/VIS using Nanodrop 2000c (Thermo Scientific) at 488 nm to quantitate the DOX loading. A calibration curve was generated, and the Lambert–Beer law was used to determine the concentration of the TBSV-loaded DOX. The drug encapsulation efficiency (EE) and loading capacity (LC) of DOX in NPs were also determined according to the following formulae:

EE(%) = Amount of DOX in the NPs/total amount of DOX added (×100)

LC(%) = Amount of DOX in the NPs/NPs weight (×100)

### 2.7. Cell Viability Assay

PPC-1 and M21 cells were plated in a white opaque 96-well plate (5000 cells/well) and incubated at 37 °C in a 5% CO_2_ atmosphere for 24 h. TBSV-RPAR^DOX^, TBSV-WT^DOX^, and free DOX were then added to the wells at a final drug concentration of 25, 5, 1, 0.2, or 0.04 μmol/mL. Empty TBSV NPs were also used at the same concentration used for DOX-loaded NPs. After 30 min incubation at 37 °C, the medium was carefully removed, and the wells were washed twice with fresh medium and allowed to grow at 37 °C in a 5% CO_2_ atmosphere. Cell viability was measured after a period of 48 h using the CellTiter-Glo reagent (Promega, Madison, USA) following the manufacturer’s protocol. The EnSight multimode plate reader was used to measure the chemoluminescence of the samples. Data are presented as mean ± SD of biological replicates (*n* = 6).

### 2.8. In Vivo Biodistribution Analysis of TBSV

The animal experiments were conducted according to protocols approved by Estonian Ministry of Agriculture, Committee of Animal Experimentation (project #159). TBSV-RPAR or TBSV-WT (200 μg in PBS) were injected into the tail vein of 8–10-week-old female Balb/c mice. Twenty-four hours after i.v. administration, the mice were perfused via the left ventricle using 20 mL of PBS. Organs were collected and fixed in cold 4% *w*/*v* paraformaldehyde in PBS at 4 °C for 24 h, washed in PBS at room temperature for 1 h, and cryoprotected in 15% *w*/*v* and 30% *w*/*v* sucrose (Sigma-Aldrich, Saint Louis, MO, USA) at 4 °C overnight. Cryoprotected and fixed tissues were frozen in optimal cutting temperature compound (OCT compound; Leica), cryosectioned at 10μm on Superfrost+ slides (Thermo Scientific, Waltham, MA, USA), and stored at −20 °C. Air-dried tissue sections were rehydrated in PBS for 10 min followed by permeabilization in PBS + 0.2% *v*/*v* Triton (Triton X-100, AppliChem, Darmstadt, Germany) at RT for 10 min. Tissue slides were washed with PBST (PBS + 0.05% *v*/*v* Tween-20 (Sigma-Aldrich, Saint Louis, MO, USA) and blocked with 1× blocking buffer (5% *w*/*v* BSA, 5% *v*/*v* FBS, and 5% *v*/*v* goat serum in PBS with 0.05% Tween-20) at RT for 1 h. Primary antibodies diluted in 0.2× blocking buffer were added and incubated at 4 °C overnight. Incubation with secondary antibodies was performed at RT for 30 min, followed by washing and nuclear counterstaining with DAPI (1 μg/mL in PBS) at RT for 8 min. TBSV NPs were detected using rabbit anti-TBSV (dilution 1:200) and Alexa Fluor 647 goat anti-rabbit antibody (dilution 1:500, Invitrogen by Thermo Fisher Scientific). The coverslips were mounted in Fluoromount-G and imaged using an Olympus FV1200MPE confocal microscope (Olympus Europa SE & Co. KG, Hamburg, Germany).

### 2.9. Statistical Analysis

All quantitative data are presented as mean ± SD, and statistical significance (p) was calculated by a two-tailed Student’s t-test. All analyses were carried out using GraphPad Software 8.4.3.

## 3. Results

### 3.1. Construction, Production, and Purification of Wild-Type (WT) and Chimeric TBSV NPs

**Figure 1:**
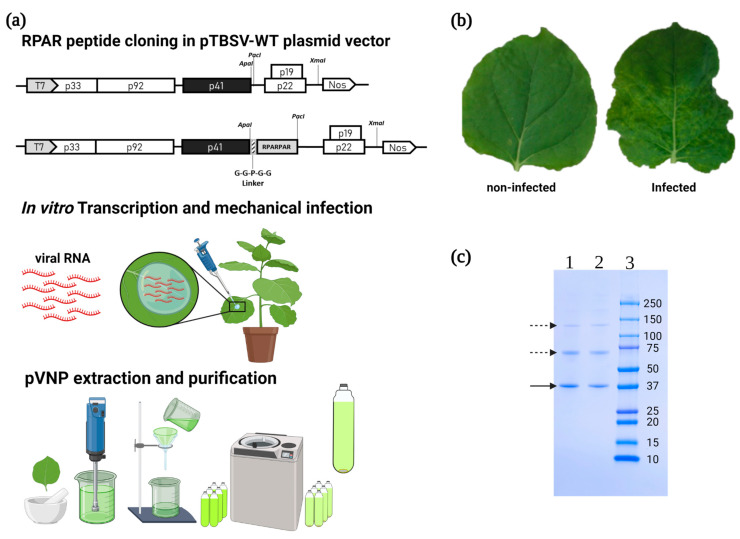
Preparation of TBSV-RPAR expression construct and recombinant virus production. Experimental flow-chart: the TBSV CP (p41) is genetically engineered by fusion of the RPAR peptide coding sequence in the pTBSV-WT plasmid vector; the modified TBSV vector is transcribed in vitro to obtain an infectious viral RNA to be used for plant inoculum; infected tissue is used for virus purification, and purified batches are controlled by SDS-PAGE. (**a**) Map of the TBSV–WT/RPAR plasmids, RNA inoculum *in planta*, and pVNP purification procedure. The five ORFs (p33 and p92 (RNA-dependent RNA polymerase), p41 (coat protein, CP), p22 (movement protein), p19 (silencing inhibitor)) are shown. T7, promoter sequence from T7 phage; Nos, terminator sequence of the *Agrobacterium tumefaciens* nopaline synthase encoding gene; *ApaI* and *PacI*, restriction sites used for RPAR CP genetic fusion; *XmaI*, restriction site used for vector linearization; striped box, GGPGG linker. (**b**) *N. benthamiana* leaves from non-infected plants (left) and after infection with TBSV-RPAR (right). (**c**) SDS-PAGE of TBSV-RPAR NPs. Purified TBSV samples (4 µg per lane) were denatured and electrophoretically separated on 4–20% Tris-glycine gel (Bio-Rad), stained with Coomassie Blue. TBSV-WT (1), TBSV-RPAR (2), Precision Plus Protein Dual Color Standards (Bio-Rad) (3). The molecular masses of the marker bands are indicated. Solid arrow indicates monomeric coat protein bands; dashed arrows indicate CP dimers and aggregates.

TBSV was genetically engineered to display the RPAR peptide as a fusion to the C-terminus of the viral CP. To avoid potential interference with CP folding and virus assembly, a GGPGG linker was inserted between the CP and the peptide (Figure 1a). The linker may also provide flexibility to facilitate the engagement of the peptide with the cell surface receptors [16,17]. Mechanical inoculation of *N. benthamiana* plants with the in vitro transcribed viral RNA resulted in chlorotic vein clearing, a typical symptom of infection (Figure 1b). The RNA extracted from infected plants was retro-transcribed, and sequencing of cDNA PCR fragments confirmed the presence of RPAR peptide and linker in frame with the genomic RNA-encoded CP. A second round of infection with tissue extracts from infected plants was carried out to confirm the genetic stability of the engineered virus. The yield of purified TBSV-RPAR and wild-type TBSV (TBSV-WT) NPs was ~1 mg/g wet weight. SDS-PAGE and Coomassie Blue staining were used to confirm the presence of the viral CP and verify the purity of preparations (Figure 1c). These results show that the exogenous CP-fused RPAR peptide does not compromise the fitness and *in planta* propagation of the TBSV.

### 3.2. Fluorophore Labeling of TBSV NPs

**Figure 2:**
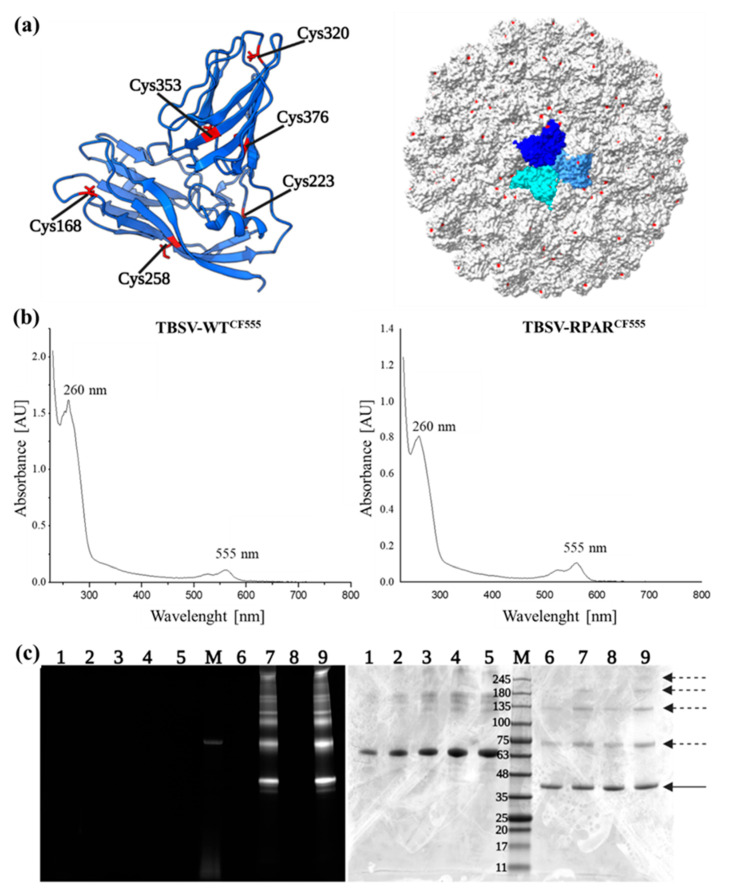
CF555 labeling of the TBSV NPs. (**a**) Structure of the CP and the TBSV virions. Left: ribbon model of the TBSV CP subunit (GeneBank U80935.1) with cysteine residues indicated. Surface-exposed cysteine residues with free sulfhydryl groups (C168, C320, and C223) are highlighted in red. Right: crystal structure of the TBSV particle. The 180 CP units are arranged in a T = 3 symmetry with the CP units in a representative trimer highlighted in shades of blue. The structural information was retrieved from https://www.rcsb.org/, accessed on 15 March 2022, Protein Data Bank file PDB:2TBV. The images were created using UCFS ChimeraX software. (**b**) UV-visible spectra of TBSV-WT and TBSV-RPAR after conjugation with CF555-maleimide. (**c**) SDS-PAGE of TBSV NPs following CF555 labeling. Gel was imaged under fluorescence channel (left) and, after Coomassie Blue staining, under white light (right). M: Opti-Protein XL molecular weight marker; lanes 1–5: 1, 2, 3, 4, and 5 µg/lane BSA; lanes 6 and 7: unlabeled and CF555-labeled TBSV-WT; lanes 8 and 9: unlabeled and CF555-labeled TBSV-RPAR. Solid arrow indicates monomeric coat protein bands; dashed arrows indicate CP dimers and aggregates.

To design a strategy for the covalent coupling of fluorophores to the TBSV capsid, the crystal structure of TBSV was examined. The lysine residues, suitable for N-hydroxysuccinimide (NHS)-ester-based bioconjugation, are on the non-accessible inner surface of the capsid and unavailable for conjugation [18,20]. In contrast, five cysteine residues, C168, C223, C320, C376, and C353 (Figure 2a), are present on the surface of the TBSV capsid. Whereas cysteines C356 and C376 form a disulfide bridge, C168, C320, and C223 have free sulfhydryl groups available for bioconjugation. Therefore, fluorescent dye functionalization was addressed by thiol-malemide bioconjugation using CF555-maleimide. TBSV NPs were incubated in the presence of 2000 molar excess of the dye per particle, followed by ultracentrifugation to remove the free CF555 and quantitation of the degree of labeling by UV-visible spectroscopy (Figure 2b). Highly efficient labeling was observed for both TBSV-WT^CF555^ (504 ± 24 dye molecules/particle) and TBSV-RPAR^CF555^ NPs (540 ± 10 dye molecules/particle). SDS-PAGE followed by fluorescence gel imaging and subsequent Coomassie Blue staining confirmed the covalent binding of the CF555 fluorophore to the NPs (Figure 2c).

### 3.3. In Vitro Binding of TBSV-RPAR to NRP-1

**Figure 3:**
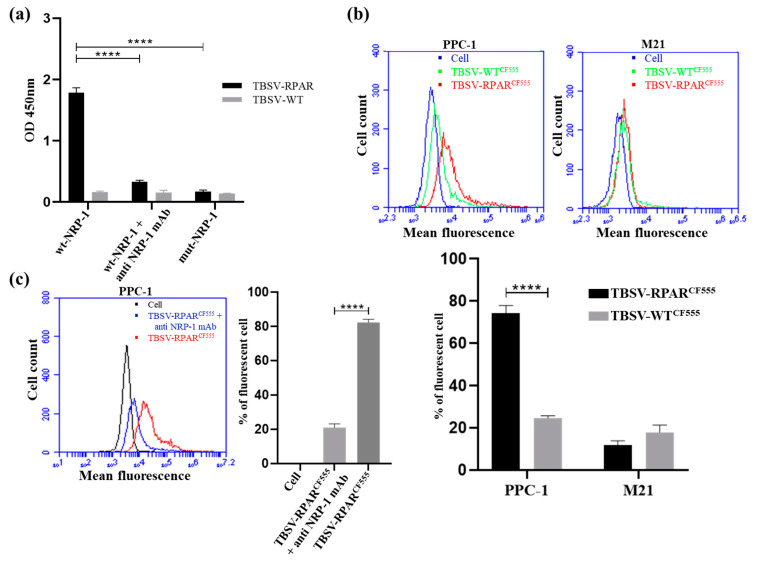
TBSV-RPAR NPs interact with recombinant NRP-1 and with cell surface NRP-1. (**a**) Binding of TBSV-RPAR and TBSV-WT to wt-NRP-1 or mut-NRP-1. TBSV detection using sandwich ELISA used primary rabbit anti-TBSV antibody followed by HRP-conjugated anti-rabbit antibody, followed by chromogenic reaction and quantitation of signal at 450 nm. CendR-blocking anti-NRP-1 antibody reduced TBSV-RPAR binding. **** *p* < 0.0001 Student’s *t*-test. (**b**) Flow cytometry of CF555-labeled TBSV-WT and TBSV-RPAR incubated with NRP-1-expressing PPC-1 and NRP-1-negative M21 cells. Cell count vs. mean fluorescence and percentage of fluorescent cells are shown. **** *p* < 0.0001. (**c**) Effect of anti-NRP-1 antibody on binding of CF555-labeled TBSV-RPAR to PPC-1 cells. Cell count vs. mean fluorescence and percentage of fluorescent cells after incubation with TBSV-RPAR^CF555^ are shown. **** *p* < 0.0001 Student’s *t*-test.

We next tested the ability of RPAR-functionalized TBSV NPs to interact with NRP-1 under cell-free conditions and with NRP-1 expressed on the cell surface. The recombinant b1b2 domain of human NRP-1 (wt-NRP-1), or triple mutant b1b2 with nonfunctional CendR binding pocket (mut-NRP-1), was coated on multiwell plates, and binding of TBSV-RPAR and TBSV-WT was assessed by sandwich ELISA. RPAR functionalization of TBSV NPs increased wt-NRP-1 binding ~11-fold. In contrast, in the wells coated with the mut-NRP-1, only baseline TBSV-RPAR binding was observed. Furthermore, preincubation of wells with a monoclonal anti-NRP-1 antibody, which interferes with the binding of the CendR ligands to NRP-1 [36], resulted in a ~5.5-fold decrease in the binding of TBSV-RPAR (Figure 3a). These cell-free binding studies confirmed the CendR-dependent NRP-1 binding of TBSV NPs.

Next, we studied the binding of WT and RPAR-coated CF555-labeled TBSV NPs to cultured NRP-1-positive PPC-1 prostate cancer cells and NRP-1-negative M21 melanoma cells. Flow cytometry demonstrated increased RPAR-dependent binding of TBSV NPs to PPC1 cells and a very low signal for both peptide-guided and control NPs in the NRP-1-negative M21 cells (Figure 3b). Preincubation of PPC1 cells with the CendR-blocking antibody decreased the binding of TBSV-RPAR ~4-fold (Figure 3c).

**Figure 4:**
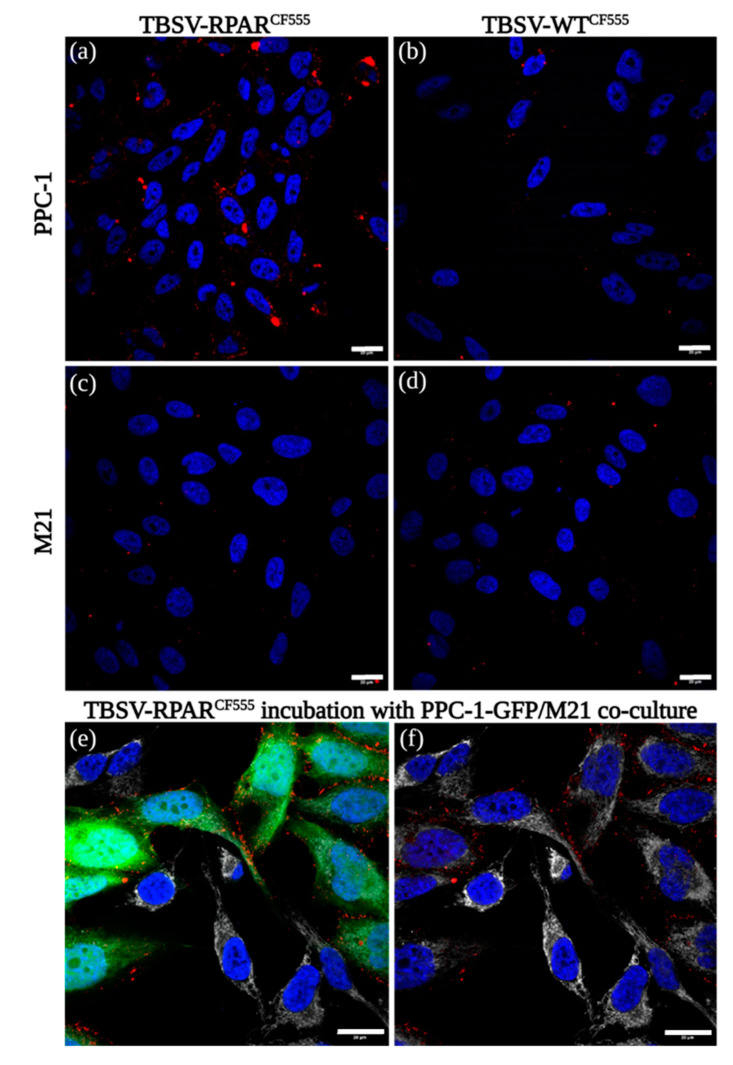
TBSV-RPAR NPs target NRP-1-expressing cells. Confocal microscopy images of PPC-1 or M21 cells incubated at 37 °C for 1 h with TBSV-RPAR^CF555^ (**a**,**c**) or TBSV-WT ^CF555^ (**b**,**d**). Red = TBSV NPs; blue = DAPI (nuclei). (**e**,**f**) TBSV-RPAR^CF555^ binding to co-culture of PPC-1-GFP and M21 cells. TBSV-RPAR^CF555^ NPs are taken up only by GFP-positive PPC-1 cells. Red = TBSV-NPs; blue = DAPI (nuclei); green = GFP expressed in PPC-1 cells; white = p32. Panel f is not showing the GFP fluorescence channel. Scale bars = 20 µm.

The binding and internalization of TBSV-WT^CF555^ and TBSV-RPAR^CF555^ in PPC-1 and M21 cells were further investigated by confocal microscope imaging. PPC-1 cells showed uptake of TBSV-RPAR^CF555^ NPs and no binding of TBSV-WT^CF555^ (Figure 4a,b). In M21 cells, no labeling was detected following incubation with both TBSV-WT^CF555^ and TBSV-RPAR^CF555^ (Figure 4c,d). After the addition of TBSV-RPAR^CF555^ to the mixed culture of Green Fluorescent Protein (GFP)-expressing PPC-1 cells (PPC-1-GFP) and “dark” M21 cells, the binding of NPs was seen only in NRP-1-positive PPC-1 cells (Figure 4e).

**Figure 5:**
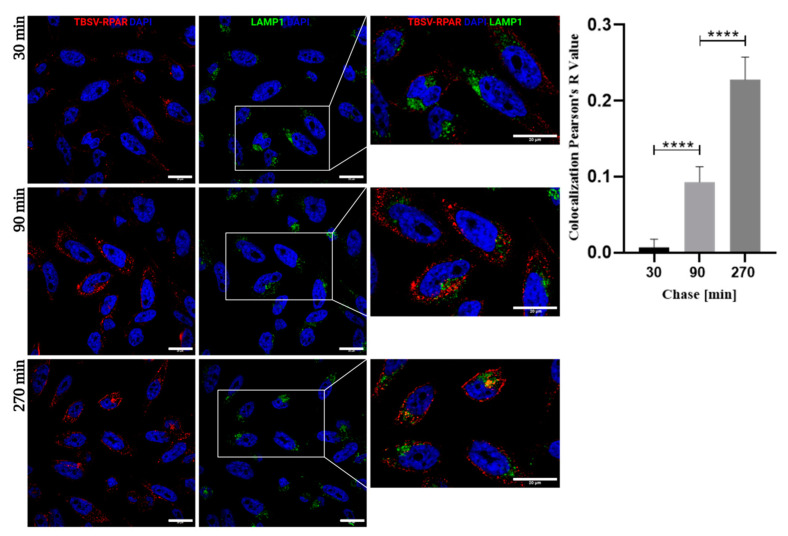
Internalized TBSV-RPAR NPs are routed to the LAMP-1-positive lysosomal compartment. PPC-1 cells were incubated with TBSV-RPAR for 30 min, washed to remove unbound NPs, and imaged at 30, 90, and 270 min time points. The cells were stained with lysosomal marker LAMP-1 (green) and anti-TBSV antibody (red). Blue: DAPI (nuclei). Scale bars = 20 µm. The relative co-localization was quantified using ImageJ software with the Pearson’s R value shown in the bar chart. **** *p* < 0.0001 (Student’s *t*-test).

In PPC-1 cells, pulse-chase co-localization studies of TBSV-RPAR with the lysosomal marker LAMP-1 showed the time-dependent entry of the peptide-guided NPs in the endolysosomal pathway (Figure 5).

### 3.4. TBSV-RPAR Loading with DOX and Cytotoxicity Test In Vitro

**Figure 6:**
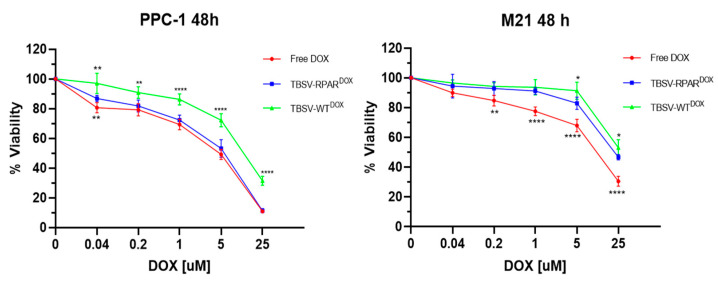
Cytotoxic effect of drug-loaded TBSV-NPs. Viability of PPC-1 and M21 cells after 48 h of incubation with different concentrations of free DOX or DOX-loaded TBSV-NPs was estimated by MTT assay. Data are presented as mean ± SD of biological replicates, *n* = 6. * *p* = 0.018; ** *p* = 0.0051; **** *p* < 0.0001. Student’s *t*-test (TBSV-RPAR^DOX^ compared with TBSV-WT^DOX^ and TBSV-RPAR^DOX^ compared with free DOX).

Having established that the TBSV-RPAR NPs engage with NRP-1 under cell-free conditions and are internalized in NRP-1-expressing cells, we next studied whether this selectivity translates into increased cytotoxicity for TBSV NPs loaded with a cytotoxic anticancer drug. Doxorubicin (DOX)-loaded TBSV-NPs were prepared using a previously developed protocol [17]. DOX was added at 5000 molar excess to the TBSV-NPs in an alkaline swelling buffer containing EDTA to loosen the viral capsid to enable entry of the drug. To entrap the drug, the compact state of the viral capsid was re-established by restoring the pH and adjusting the Ca^2+^ and Mg^2+^ concentrations. Free DOX was removed from the TBSV samples by a sucrose cushion ultracentrifugation. A similar degree of DOX loading was achieved for both types of NPs: 48 ± 12 ng DOX/μg TBSV-WT and 58 ± 6 ng DOX/μg TBSV-RPAR, corresponding to 729 ± 181 and 892 ± 92 DOX molecules/virion, respectively. The encapsulation efficacy (EE) was 29 and 34%, while the loading capacity (LC) was 4.8 and 5.8%, respectively. For cellular viability assessment, PPC-1 and M21 cells were incubated with DOX-loaded TBSV NPs and free DOX, and after 48 h incubation, the cells were subjected to MTT assay (Figure 6). In PPC1 cells, TBSV-RPAR^DOX^ and free DOX were significantly more toxic than TBSV-WT^DOX^. In contrast, in M21 cells, the effect of TBSV-WT^DOX^ and TBSV-RPAR^DOX^ on cell viability was similar and significantly lower than the effect of free DOX. These data show that functionalization of the cytotoxic compound-carrying TBSV with the CendR peptide increases the toxic effect of the particles on NRP-1-positive cells.

### 3.5. Systemic TBSV-RPAR NPs Home to Pulmonary Tissue

**Figure 7:**
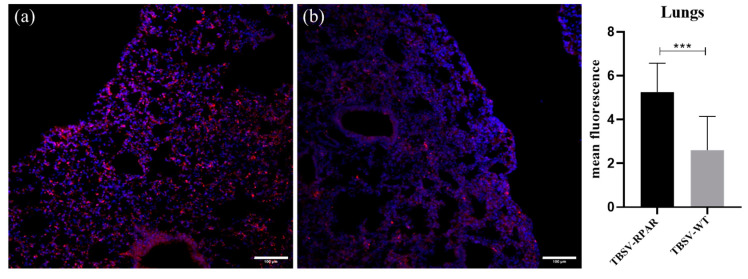
TBSV-RPAR accumulation in the pulmonary tissue following i.v. administration. Immunofluorescent localization of TBSV in lung sections of mice that received i.v. injection with (**a**) TBSV-RPAR and (**b**) TBSV-WT. Blue: DAPI (nuclei). Scale bars = 100 μm. TBSV fluorescence signal (pink) was quantified using ImageJ software and expressed as mean of fluorescence. Five randomly chosen fields from 3 different animals per group were analyzed. Data represent mean ± SD, *** *p* < 0.001 (Student’s *t*-test).

NRP-1 is widely expressed in normal tissues and organs, including on the luminal side of the vascular endothelium. Published studies have shown that after systemic injection, NPs guided with CendR peptides show a robust homing to the first-met microvasculature in the pulmonary and cardiac tissues [27,37]. To study whether CendR peptides have similar activity when present on TBSV NPs, the biodistribution of TBSV-WT and TBSV-RPAR in the Balb/c mice following intravenous (i.v.) administration and 24 h circulation was studied. Confocal imaging of TBSV immunoreactivity showed a robust signal in the pulmonary tissue of TBSV-RPAR-injected mice and a lower amount of NPs in the lungs of TBSV-WT-injected mice (Figure 7).

These data show that RPAR-targeted TBSV NPs show a lung tropism similar to that of other classes of NPs tested in the past.

## 4. Discussion

Here, we report proof-of-concept studies on the targeting of TBSV NPs with prototypic CendR peptide, RPAR. We show that the display of RPAR as a fusion to the viral CP increases the binding of TBSV NPs to recombinant NRP-1, results in their uptake in cultured NRP-1-positive cells, and, following systemic administration, promotes their accumulation in the lung microvasculature. We also show cargo loading of the TBSV NPs using two strategies: chemical conjugation to the exposed free cysteine residues on the surface of the viral capsid via sulfhydryl–maleimide coupling, and a diffusion-based strategy for drug loading into the internal cavity of the viral NPs. Collectively, our studies show the feasibility of the TBSV-derived precision-guided nanocarriers for the delivery of imaging agents and therapeutic compounds. In recent years, TBSV has emerged as a versatile drug delivery platform. The TBSV capsid is stable and remains intact after incubation with human serum at 37 °C [38]. Uniform batches of TBSV NPs can be purified from infected plants using a well-established protocol. Upon systemic administration, TBSV is present in the bloodstream for a longer time (still detectable at 7 days) as compared to PVX (disappeared after 48 h) [39]. TBSV NPs can be functionalized through chemical modifications, osmotic loading techniques, and genetic engineering [16]. TBSV has been employed as a drug delivery platform for medulloblastoma [17]. In this preclinical study, doxorubicin-loaded TBSV NPs genetically modified to display tumor-homing peptides on their external surface were shown to exert antitumor activity in vitro and accumulate in the malignant lesions. A potential disadvantage of all plant viruses is that they are generally immunogenic, a challenge that can be addressed by coating the carriers with shielding compounds such as polyethylene glycol (PEG) [40,41]. We chose to use homing peptides as TBSV-guiding modules due to their emerging translational relevance. Homing peptides are small (<9 amino acids), show low immunogenicity, are not species-specific, and have affinity in the range avoiding “binding site barrier” known to limit extravasation and tissue penetration of high-affinity ligands such as antibodies [42]. In particular, the CendR targeting module used in the current study for TBSV targeting is present in all tumor-penetrating peptides (TPPs), an emerging class of tumor-homing peptides with the ability to extravasate and penetrate throughout tumor parenchyma. Whereas the CendR peptides have been demonstrated to enable targeting of biological phage NPs and different synthetic nanoscale scaffolds [43,44,45,46], the structural and physicochemical properties of NPs can have a dramatic effect on targetability with affinity ligands. Here, we show that RPAR peptide remains active when displayed on the TBSV surface as a C-terminal fusion to the CP. TBSV-RPAR binds to recombinant NRP-1 under cell-free conditions, is internalized in cultured NRP-1-positive cells, and homes to lung microvessels following i.v. administration. The implication of these observations is the applicability of the entire TPP family for precision targeting of TBSV NPs, a possibility that remains to be rigorously tested in follow-up studies. The homing peptides were displayed on the TBSV NPs at 180 copies/32 nm particle (~3.4-fold higher density than in the case of 60 nm T7 bacteriophage used for homing peptide discovery at ~200 peptides/60 nm particle). Targeting ligand density is a critical factor affecting the NP recruitment to the target cells and a key determinant of the choice of cellular internalization pathway [47]. Somewhat counterintuitively, higher ligand density does not always result in a more effective targeting [48]. The effect of the relationship between the density of the targeting peptides on the TBSV surface and the targeting selectivity remains to be tested in the follow-up studies. A majority of nanocarriers are taken up by cells via endocytosis and are primarily routed towards lysosomes [49]. In our study, TBSV-RPAR NPs were also internalized via the endolysosomal uptake pathway. Lysosomal trafficking has been reported for other plant viruses such as the CPMV [50], Physalis Mottle Virus (PhMV) [51], and Sesbania Mosaic Virus (SeMV) [52], which, even if belonging to different taxonomic families, have shapes and sizes similar to those of TBSV. Whereas the lysosomal delivery of nanocarriers may be useful in the case of certain classes of therapeutics (e.g., drugs for the treatment of lysosomal storage disorders), the harsh milieu of the lysosomal compartment due to low pH and an array of hydrolases capable of breaking down all types of biological polymers (proteins, nucleic acids, carbohydrates, and lipids) represents a barrier to drug delivery. Therefore, strategies aimed at facilitating the endosomal escape of the NPs [53], or their routing to different compartments, are favored. Recently, we showed that NRP-1 interacts with endosomal SNX-BAR sorting complex promoting exit 1 (ESCPE-1) and that this interaction mediates retrograde trafficking of NPs functionalized with a CendR peptide [54]. Optimization of the TBSV-CendR NPs for preferential use of the retrograde trafficking pathway (e.g., by modulating the density of surface peptides) and/or employing endosomal escape-promoting strategies (such as adding poly-histidines as endosolytic buffering agents) may prove useful in preventing lysosomal routing.

## 5. Conclusions

Our study demonstrates that CendR peptide-displaying TBSV NPs can specifically interact with the NRP-1 receptor, actuate cellular binding and internalization in cultured cells, and display lung homing/penetration in vivo. Results confirm the versatility of TBSV to be efficiently modified by different strategies, i.e., genetic engineering, cargo loading, and chemical bioconjugation, opening the way to a plethora of different applications in the nanomedicine field. In particular, CendR-mediated targeting could be used for the development of TBSV-based diagnostic and therapeutic platforms for pulmonary targeting and solid tumor delivery.

## Data Availability

Not applicable.
